# The Effectiveness of Digital Intervention on Psychological Resilience in Postoperative Breast Cancer Patients During Chemotherapy Intervals: Quasi-Experimental Study

**DOI:** 10.2196/81610

**Published:** 2026-09-11

**Authors:** Xin He, Qianyu Zhang, Liping Wang, Qunying Fang, Chen Chen, Shujin Wang, Erlong Sun, Jinghan Yang, Weichao Liu

**Affiliations:** 1 School of Public Health and Nursing, Hangzhou Normal University, No. 2318 Yuhangtang Road, Hangzhou Normal University Cangqian Campus, Hangzhou, Zhejiang, 311121, China, 86 57128861971; 2 Engineering Research Center of Mobile Health Management System, Ministry of Education, Hangzhou, Zhejiang, China; 3 Zhejiang Cancer Hospital, Hangzhou Institute of Medicine (HIM), Chinese Academy of Sciences, Hangzhou, Zhejiang, China; 4 Nanchang Institute of Technology, Nanchang, Jiangxi, China

**Keywords:** breast cancer, chemotherapy intervals, digital intervention, psychological resilience, mindfulness based cancer recovery, resilience model for breast cancer

## Abstract

**Background:**

Patients with breast cancer during postoperative chemotherapy intervals commonly experience psychological distress and reduced resilience while recovering at home. Digital mindfulness interventions may provide accessible psychological support during this vulnerable period; however, evidence regarding tailored interventions for postoperative patients with breast cancer during chemotherapy intervals remains limited.

**Objective:**

This study aimed to examine the effectiveness of a digital intervention on psychological resilience in postoperative patients with breast cancer during chemotherapy intervals.

**Methods:**

A quasi-experimental study with repeated measures was conducted from October 2021 to June 2022. A total of 80 eligible participants were recruited from the Department of Breast Surgery at a tertiary hospital in Zhejiang Province, China, and 71 completed the study. The control group received routine discharge instructions and nursing follow-ups, whereas the intervention group additionally received an 8-week digital psychological resilience intervention. Outcomes were assessed at baseline (T0), 3 months post intervention (T1), and 6 months post intervention (T2). The measures included the Connor-Davidson Resilience Scale (CD-RISC), Hospital Anxiety and Depression Scale (HADS), Social Support Rating Scale (SSRS), Breast Cancer Survivor Self-Efficacy Scale (BCSSS), and Functional Assessment of Cancer Therapy-Breast (FACT-B). Independent-samples *t* tests, chi-square tests, and repeated-measures ANOVA were performed using SPSS (version 26.0; IBM Corp).

**Results:**

No statistically significant baseline differences were observed between the two groups in the outcome measures. At T1, the intervention group had higher CD-RISC scores than the control group (mean 67.58, SD 11.41 vs mean 62.09, SD 10.18; *P*=.036) and higher BCSSS scores (mean 42.36, SD 3.59 vs mean 39.23, SD 4.90; *P*=.003). However, these between-group differences were no longer statistically significant at T2 (*P*>.05). Significant time effects and group×time interaction effects were observed for both psychological resilience and self-efficacy (*P*<.05). HADS scores were lower in the intervention group than in the control group at both T1 (mean 11.78, SD 1.48 vs mean 15.86, SD 1.54; *P*<.001) and T2 (mean 9.69, SD 1.51 vs mean 12.03, SD 1.42; *P*<.001), with significant time, group, and group×time interaction effects (*P*<.05). For SSRS and FACT-B scores, no statistically significant between-group differences were found at T1 or T2 (*P*>.05), although both scales showed significant time effects (*P*<.05).

**Conclusions:**

The digital psychological resilience intervention effectively alleviated anxiety and depression in postoperative patients with breast cancer during chemotherapy intervals. It also significantly enhanced their psychological resilience and self-efficacy in the early postintervention phase. Although the improvements in psychological resilience and self-efficacy did not remain statistically superior to those of the control group at the 6-month endpoint, the intervention improved the recovery trajectory of the patients and provided significant short-term benefits during the early postintervention period. Future research should explore strategies to maintain these gains over the long term.

## Introduction

According to the Global Cancer Statistics 2022 report, female breast cancer accounted for approximately 2.309 million new cases worldwide, representing 23.8% of all female cancer diagnoses, with incidence rates continuing to increase annually [1,2]. Approximately 81.4% of postoperative patients with breast cancer require 4‐8 chemotherapy cycles [3,4]. Typically, a 2- to 3-week interval is required between chemotherapy cycles to allow patients to recover from treatment-related toxicities, during which patients predominantly convalesce at home [5]. The combined physiological and psychological burden of cancer diagnosis, surgery, and chemotherapy results in significant mental health morbidity, with anxiety and depressive disorders affecting up to 74% of patients with breast cancer during chemotherapy intervals [6,7].

Psychological resilience, also referred to as resilience, is defined as a dynamic process through which individuals actively cope with stress or adversity, and successfully adapt under the interplay of risk and protective factors [8]. Research indicates that individuals with higher levels of resilience are more capable of managing negative emotions such as anxiety and depression, thereby alleviating psychological distress and improving overall health outcomes [9]. Notably, resilience is not a fixed inherent trait, but a dynamic and modifiable capacity that can be enhanced through targeted psychological or behavioral interventions [8]. Mindfulness-Based Cancer Recovery (MBCR) is an increasingly adopted psychological intervention in cancer patients, which integrates meditation and yoga practices to alleviate anxiety and depressive symptoms by enhancing psychological resilience [10]. Patients with breast cancer during chemotherapy intervals commonly experience cancer-related fatigue and immunocompromised status [11-13]. While the conventional in-person group-based MBCR model effectively enhances psychological resilience and alleviates anxiety and depression symptoms, it concurrently imposes physical challenges, infection risks, and financial burdens on patients. These limitations were significantly amplified during the COVID-19 pandemic.

Digital intervention technology can overcome limitations related to time and space, allowing patients to complete MBCR anytime and anywhere, thereby reducing the risks and burdens associated with in-person interventions. Wang et al [14] conducted a 4-week online MBCR program incorporating mindfulness meditation, body scan, and mindful breathing exercises for 51 patients with breast cancer. Their findings suggested improvements in symptom burden and quality of life. Lengacher et al [15] implemented a 6-week smartphone app-based MBCR program for 13 breast cancer survivors, incorporating sitting meditation, walking meditation, body scans, and mindful yoga. The intervention was associated with reductions in anxiety, depressive symptoms, and perceived stress. Rezaee et al [16] developed a mobile app (CaRA) for patients with breast cancer to enhance psychological resilience and quality of life and conducted learnability and usability testing among users.

However, existing digital mindfulness studies have not specifically targeted postoperative patients with breast cancer during chemotherapy intervals. These studies failed to adequately address the unique characteristics of this population, particularly upper limb functional limitations secondary to surgical incisions, lymphedema, and medical devices such as peripherally inserted central catheter (PICC) lines and implantable venous access ports. These factors significantly compromise the feasibility of current digital mindfulness interventions for this patient group. Furthermore, limited attention has been paid to evaluating digital mindfulness interventions from the perspective of changes in psychological resilience.

In view of this, the present study aimed to develop an online MBCR program tailored for postoperative patients with breast cancer during chemotherapy intervals and to evaluate the effectiveness of this digital intervention in enhancing psychological resilience within this vulnerable population.

## Methods

### Research Design

This study was conducted between October 2021 and June 2022 in two breast surgery wards (A and B) of a tertiary hospital in Zhejiang Province, China. We recruited patients with breast cancer undergoing their first course of postoperative chemotherapy. Using a nonrandomized, quasi-experimental design, patients from Ward A were assigned to the control group, and those from Ward B were assigned to the intervention group after obtaining informed consent. The nonrandomized design was adopted due to practical considerations: the digital intervention was implemented as part of an online case management system. Patients within the same ward frequently interacted online, making allocation concealment and prevention of between-group contamination unfeasible [17].

### Participants

Inclusion criteria were as follows: (1) age of ≥18 years, (2) pathologically confirmed diagnosis of breast cancer, (3) ability to read and write in Chinese, (4) access to a smartphone (either the patient or primary caregiver), and (5) voluntary participation with signed informed consent. Exclusion criteria were as follows: (1) patients unaware of their condition, (2) patients with physical disabilities affecting participation, or (3) patients with a history of psychological disorders or psychiatric illnesses.

### Sample Size

The sample size was determined using repeated-measures ANOVA for quantitative data, with the following formula:


$$n=\frac{2\sigma^{2}{\left(Z_{1-\alpha /2}+Z_{1-\beta }\right)}^{2}}{\delta^{2}}\left\{\left[\frac{1}{r}+\left(1-\frac{1}{r}\right)\rho \right]-\frac{\rho^{2}}{\frac{1}{p}+\left(1-\frac{1}{p}\right)\rho }\right\}$$

A two-sided significance level of α=.05 and a statistical power of 80% were used. The sample size calculation was based on the primary outcome, namely the CD-RISC total score. Based on published postintervention psychological resilience scores measured using the CD-RISC [18] (control group: mean 54.55, SD 8.95; intervention group: mean 60.21, SD 9.50), the calculated required sample size was approximately 34 participants per group. Considering a potential 10%‐20% dropout rate, we ultimately enrolled a total of 80 participants, with 40 participants in each group.

### Intervention Framework and Design

#### Theoretical Framework

**Figure 1.**
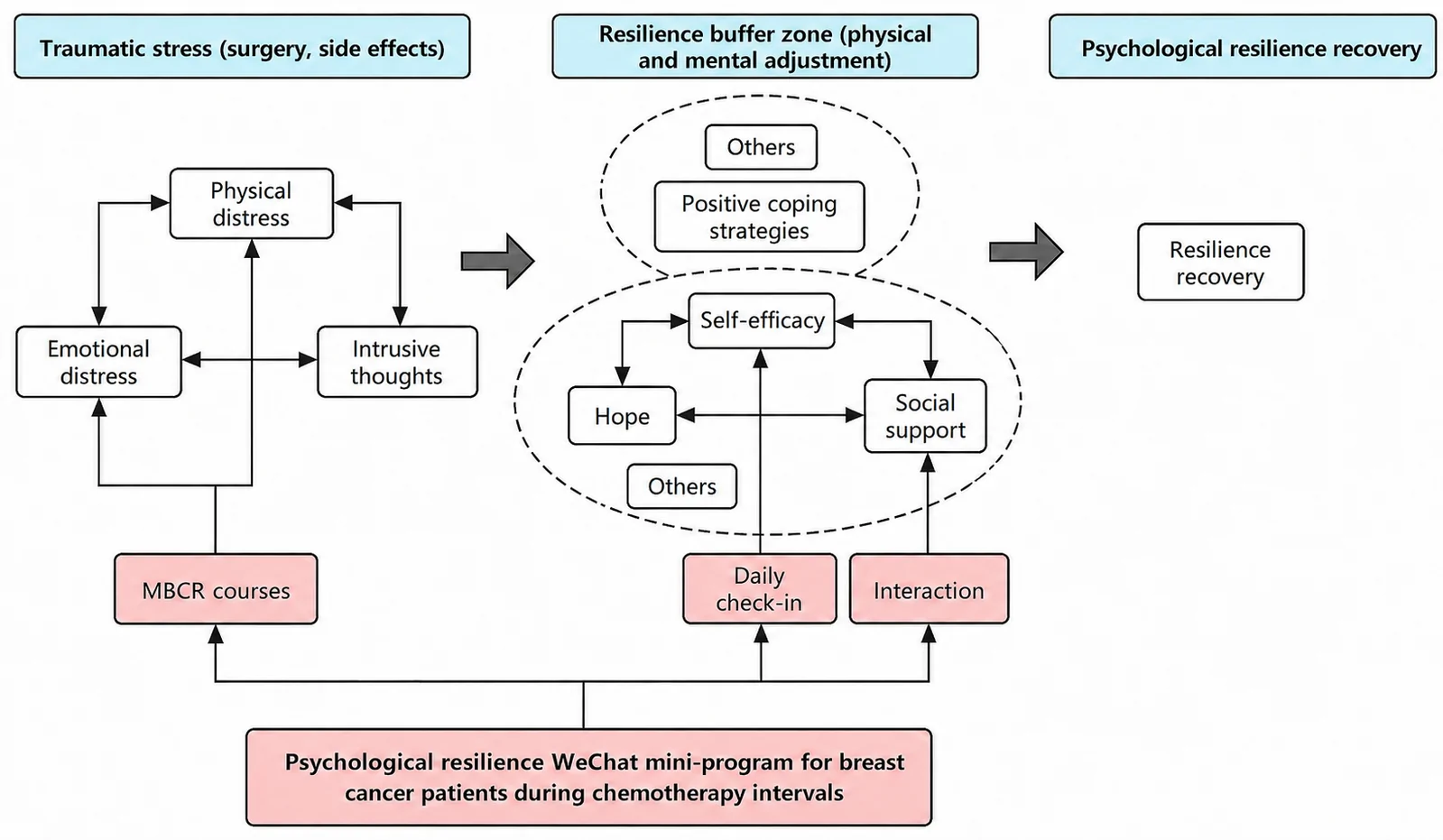
Correspondence between the RM-BC model and the psychological resilience WeChat mini-program.

This study developed the digital intervention based on Ye Zengjie’s Resilience Model for Breast Cancer (RM-BC) [19,20]. The model proposes that the dynamic interaction between risk factors and protective factors influences patients’ psychological resilience. By controlling risk factors (eg, physiological distress, emotional distress, and intrusive thoughts) and enhancing protective factors (eg, social support, self-efficacy, positive coping strategies, and hope), the psychological resilience of patients with breast cancer can be improved [19], ultimately alleviating anxiety and depression. The digital intervention was implemented via a WeChat mini-program with specific functional modules, including MBCR courses (mindfulness yoga, mindfulness meditation, and breathing exercises), interaction, and daily check-in. The relationship between each module of the WeChat mini-program and the protective and risk factors is detailed in Figure 1.

#### Mindfulness Course Design

For this study, we developed an online mindfulness-based cancer recovery program tailored for postoperative patients with breast cancer during chemotherapy intervals. The intervention was designed by a panel consisting of one psycho-oncology specialist, three breast cancer nursing experts, and one breast cancer medical specialist. The curriculum was adapted from Mindfulness-Based Cancer Recovery (developed by Canadian psychosocial oncology experts Linda E Carlson and Michael Speca), with modifications to address the specific needs of postoperative patients with breast cancer during chemotherapy intervals [21].

#### Mindfulness Course Schedule

Considering the postoperative recovery process and chemotherapy needs of patients with breast cancer, the first five weeks of the thematic intervention primarily involved 20-minute mindfulness meditation and breathing exercises. The sixth and seventh weeks focused on mindfulness yoga, while the eighth week incorporated a blended practice designed to alleviate cancer-related symptoms.

In weeks 1‐5, the 20-minute guided meditation and breathing exercises aimed to anchor attention to breath and somatic sensations, enabling participants to regulate body responses with precision, alleviate negative emotions, and cultivate resilience, including mini-breathing practices, stress-response meditation, mindful awareness, and seated meditation. In weeks 6‐7, the intervention transitioned to mindful movement, featuring standing and supine yoga. Each 30-minute session comprised a 5-minute warm-up, 20-minute yoga postures, and a 5-minute cool-down. This design balanced physical activation with mindful relaxation. In week 8, the symptom-specific hybrid practices included meditation for body image distress and chemotherapy-induced nausea, breathing techniques for sleep disturbances, and yoga for cancer-related fatigue.

#### Mindfulness Yoga Adaptation

Based on expert recommendations, the yoga poses were modified to accommodate the specific needs of postoperative patients with breast cancer with PICC lines or implanted ports, as well as restricted limb mobility. The adaptations were as follows: (1) to avoid potential displacement of PICC lines or port-related complications, poses involving excessive arm elevation (poses 5‐36, 5‐39 to 5‐41, 5‐43, 5‐45 to 5‐47 in the original sequence [21]) were eliminated. (2) Due to postoperative precautions against compression of the affected limb, supine poses exerting pressure on the limbs (poses 5‐6, 5‐9 to 5‐14, and 5‐16 to 5‐22 [21]) were removed. (3) Safety protocol integration in yoga practice. A standardized safety briefing was incorporated at the initiation of each yoga session, with particular emphasis on movements that could potentially affect the affected upper limb. For all poses involving upper extremity engagement, participants received repeated verbal cues. For example, "Elevate your arm only to a comfortable height where mild stretching sensation is perceived as normal. Immediately discontinue the movement if you experience any pain or discomfort.”

#### Study Procedures

During hospitalization, licensed psychotherapists conducted biweekly conventional in-person group mindfulness sessions in the ward’s demonstration room to facilitate patients’ understanding and first-hand experience of mindfulness-based interventions.

#### Control Group

On the day of discharge, case management nurses provided both patients and their primary caregivers with standard discharge instructions. The instruction covered wound care management, potential adverse effects of chemotherapy, postoperative functional exercises, dietary recommendations, and disease monitoring. Additionally, case management nurses conducted weekly telephone follow-ups to assess patients’ status, provided health consultations, and reminded follow-up visits.

#### Intervention Group

In addition to standard discharge instructions and follow-ups, participants in the intervention group received a digital resilience intervention developed by the research team. On the day of discharge, trained research assistants (QF and ES) provided individualized instruction to both patients and their caregivers on how to use the WeChat mini-program. Participants were required to complete daily mindfulness exercises according to the mindfulness course schedule, with a minimum of four practice check-ins per week.

To ensure learning effectiveness, the mini-program required each practice session to last at least 20 minutes before a check-in could be completed; check-ins could not be completed if the practice duration was less than 20 minutes. In order to encourage adherence, the WeChat mini-program automatically sent a “check-in reminder” to patients at 8 AM daily, followed by another reminder at 8 PM for those who had not completed their check-in. If a patient failed to check in for 3 consecutive days, the system would notify the researchers, who would then contact the patient individually to identify the reason and provide assistance.

During the intervention period, patients could also engage in peer interactions through the mini-program’s “Interaction” module. They shared experiences and insights from their home-based mindfulness practice and discussed self-health management issues during the chemotherapy intervals.

### Instruments

#### General Information Questionnaire

The questionnaire consisted of 2 sections: general demographic data and disease-related information, including gender, age, education level, disease stage, surgical approach, and other relevant details.

#### Connor-Davidson Resilience Scale

The Chinese version of the Connor-Davidson Resilience Scale (CD-RISC) was used to assess psychological resilience [22]. The scale comprises 25 items across three dimensions: tenacity, strength, and optimism. Each item is rated on a 5-point Likert scale, and the total score ranges from 0 to 100, with higher scores indicating greater psychological resilience. The Chinese version of the CD-RISC demonstrated good internal consistency, with a reliability coefficient of 0.91 [22].

#### Hospital Anxiety and Depression Scale

The Chinese version of the Hospital Anxiety and Depression Scale (HADS) was used to assess anxiety and depression. This scale consists of 14 items, including 7 items for anxiety and 7 items for depression. The overall Cronbach α coefficient was 0.806, with subscale coefficients of 0.83 for anxiety and 0.82 for depression. The test-retest reliabilities of the anxiety and depression subscales were 0.921 and 0.932, respectively [23-25]. Responses are scored on a 4-point Likert scale ranging from 0 to 3, with higher scores indicating more severe symptoms [23,24].

#### Social Support Rating Scale

The Social Support Rating Scale (SSRS), developed by Xiao Shuiyuan, was used to assess social support. This 10-item scale includes three dimensions: subjective support, objective support, and support utilization. The Cronbach α coefficient was 0.92, and the test-retest reliability was 0.91 [26]. The total score ranges from 12 to 66, with higher scores indicating greater social support [27].

#### Breast Cancer Survivor Self-Efficacy Scale

The Chinese version of the 11-item Breast Cancer Survivor Self-Efficacy Scale (BCSSS) was used to assess self-efficacy [28]. The Cronbach α coefficient was 0.82. Items are rated on a 5-point Likert scale ranging from 1 (“strongly disagree”) to 5 (“strongly agree”), with higher scores indicating higher levels of self-efficacy [28].

#### Functional Assessment of Cancer Therapy-Breast

The Chinese version of the Functional Assessment of Cancer Therapy-Breast (FACT-B) was used to assess quality of life. This scale comprises 5 dimensions: physical well-being, social and family well-being, emotional well-being, functional well-being, and breast cancer–specific additional concerns [29-31]. The Cronbach α coefficient was 0.91 [29,30]. Responses are scored on a 5-point Likert scale ranging from 0 (“not at all”) to 4 (“very much”), with higher scores indicating better quality of life [29-31].

### Data Collection

A researcher (QZ) blinded to group allocation performed data collection at three predetermined time points: baseline (T0, on discharge day following the first postoperative chemotherapy cycle), 3-month follow-up (T1), and 6-month follow-up (T2).

### Statistical Analysis

Statistical analyses were conducted using Statistical Package for the Social Sciences 26.0 (SPSS 26.0; IBM Corp). Continuous variables were presented as mean and SD or median and interquartile range according to the normality of distribution, whereas categorical variables were described as frequencies and percentages. Between-group comparisons were performed using independent-samples *t* tests for continuous variables and chi-square tests for categorical variables. Longitudinal changes in psychological resilience, anxiety, and depression, social support, self-efficacy, and quality of life across the three time points were analyzed using repeated-measures ANOVA. Cohen *d* was calculated using the pooled SD to quantify the magnitude of between-group differences at each time point.

### Ethical Considerations

This study was approved by the hospital’s Ethics Committee (Approval No.: IRB-2020‐408). Written informed consent was obtained from all participants before data collection. Participant data were anonymized and kept confidential, and no identifiable personal information is reported in this manuscript. Participants did not receive financial compensation for participation. The study was prospectively registered with the Chinese Clinical Trial Registry (Registration No.: ChiCTR2100047251).

## Results

### Participant Attrition

**Figure 2.**
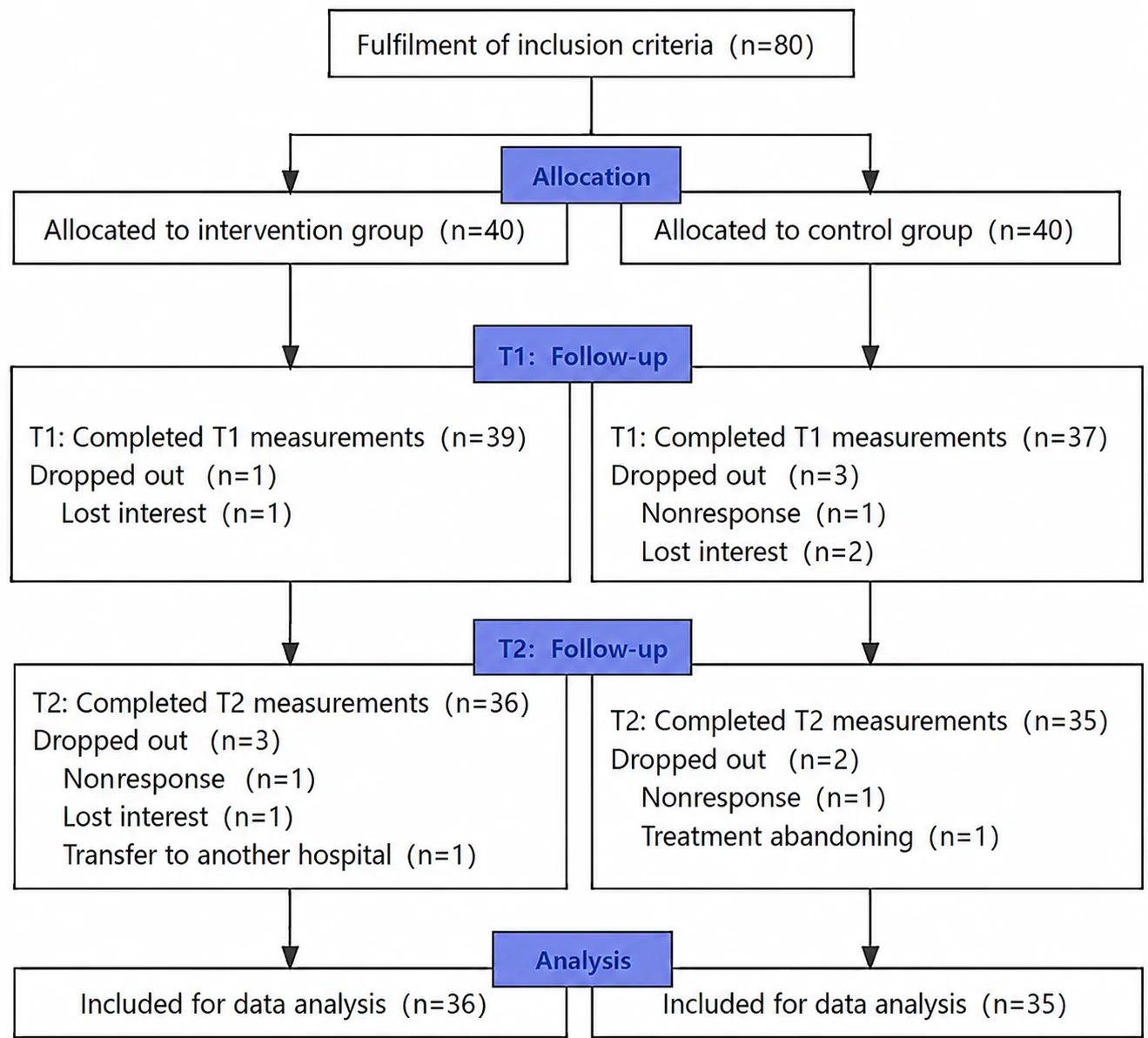
Participant attrition flowchart.

Initially, 80 eligible patients with breast cancer during their postoperative chemotherapy intervals were enrolled. Ultimately, 71 participants completed the study (intervention group: n=36; control group: n=35). The attrition details were as follows. At the T1 assessment, 1 participant in the intervention group withdrew due to loss of interest, and 3 participants in the control group withdrew (1 due to nonresponse and 2 due to loss of interest). At the T2 assessment, 3 participants in the intervention group withdrew (1 due to nonresponse, 1 due to loss of interest, and 1 due to hospital transfer), while 2 participants in the control group withdrew (1 due to nonresponse and 1 due to treatment discontinuation). The details of participant attrition are presented in Figure 2.

### Comparison of Baseline Characteristics Between the Two Groups

There were no statistically significant differences between the two groups in baseline sociodemographic or clinical characteristics (*P*>.05; Table 1).

**Table 1.** Baseline characteristics of the two groups.

Sociodemographic and clinical characteristics	Intervention group (n=36）	Control group (n=35）	Statistics	*P* value
Age, mean (SD; in years)	41.11 (8.56)	41.74 (5.64)	60.744 (−0.368)^a^	.71
Sex, n (%)				
Male	1 (2.8）	0 (0.0）	1 (0.981)^b^	.32
Female	35 (97.2）	35 (100）	—^c^	—
Residence, n (%)				
Urban	27 (75.0）	31 (88.6）	1 (2.2)^b^	.14
Rural	9 (25.0）	4 (11.4）	—	—
Religious belief, n (%)				
None	34 (94.4）	34 (97.1）	1 (0.3)^b^	.57
Yes	2 (5.6）	1 (2.9）	—	—
Marital status, n (%)				
Unmarried	2 (5.6）	0 (0.0）	1 (2.001)^b^	.16
Married	34 (94.4）	35 (100.0）	—	—
Education level, n (%)				
Primary or below	2 (5.6）	1 (2.9）	4 (6.505)^b^	.16
Junior high	15 (41.7）	16 (45.7）	—	—
Senior high	7 (19.4）	1 (2.9）	—	—
Associate degree	3 (8.3）	7 (20.0）	—	—
Undergraduate or above	9 (25.0）	10 (28.6）	—	—
Employment status, n (%)				
Unemployed	20 (55.6）	19 (54.3）	2 (1.658)^b^	.44
Employed	11 (30.6）	14 (40.0）	—	—
Retired	5 (13.9）	2 (5.7）	—	—
Living arrangement, n (%)				
Living alone	1 (2.8）	0 (0.0）	3 (2.463)^b^	.48
With spouse	30 (83.3）	33 (94.3）	—	—
With children	2 (5.6）	1 (2.9）	—	—
Other	3 (8.3）	1 (2.9）	—	—
Primary caregiver, n (%)				
Self-care	4 (11.1）	2 (5.7）	3 (3.068)^b^	.38
Spouses	24 (66.7）	29 (82.9）	—	—
Children	4 (11.1）	1 (2.9）	—	—
Other	4 (11.1）	3 (8.6）	—	—
Monthly income per capita (CNY), n (%)				
<3000	6 (16.7）	1 (2.9）	2 (4.449)^b^	.11
3000‐5000	12 (33.3）	17 (48.6）	—	—
>5000	18 (50.0）	17 (48.6）	—	—
Payment method, n (%)				
Employee insurance	20 (55.6）	23 (65.7）	2 (0.787)^b^	.68
Residents insurance	13 (36.1）	10 (28.6）	—	—
Commercial insurance	0 (0.0）	0 (0.0）	—	—
Out-of-pocket payment	3 (8.3）	2 (5.7）	—	—
Clinical stage, n (%)				
I	6 (16.7）	2 (5.7）	2 (2.217)^b^	.33
II	18 (50.0）	21 (60.0）	—	—
III	12 (33.3）	12 (34.3）	—	—
IV	0 (0.0）	0 (0.0）	—	—
Regional lymph node metastasis, n (%)				
Present	26 (72.2）	26 (74.3）	1 (0.039)^b^	.84
Absent	10 (27.8）	9 (25.7）	—	—
Chemotherapy protocol, n (%)				
AC^d^	0 (0.0）	2 (5.7）	4 (4.578)^b^	.33
EC^e^	4 (11.1）	8 (22.9）	—	—
TC^f^	8 (22.2）	8 (22.9）	—	—
AC-T/P^g^	5 (13.9）	3 (8.6）	—	—
EC-T/P^h^	19 (52.8）	14 (40.0）	—	—

a *t *test (*df*).

b Chi-square (*df*)

c Not applicable.

d AC: Adriamycin+Cyclophosphamide.

e EC: Epirubicin+Cyclophosphamide.

f TC: Taxotere+Cyclophosphamide.

g AC-T/P: Adriamycin+Cyclophosphamide-Taxotere/Paclitaxel .

h EC-T/P: Epirubicin+Cyclophosphamide-Taxotere/Paclitaxel.

### Repeated-Measures ANOVA of Outcome Measures

The total scores and subscale scores of resilience, anxiety, depression, social support, self-efficacy, and quality of life at three time points (T0, T1, and T2) for both groups followed a normal distribution but violated the sphericity assumption (*P*<.05). Thus, the Greenhouse-Geisser correction was applied. Cohen *d* values for the between-group comparisons at each time point are presented in Tables 2-6.

**Table 2.** Comparison of CD-RISC^a^ scores between the two groups (mean and SD)^b^.

Items and group	T0	T1	T2	Statistics					
				Time effect		Group effect		Group×time	
				*F* test (*df*)	*P* value	*F* test (*df*)	*P* value	F test (*df*)	*P* value
Total resilience									
Intervention, mean (SD)	63.03 (9.22)	67.58 (11.41)	68.75 (9.34)	6.788 (1.408, 97.123	.005	0.751 (1, 69)	.39	7.595 (1.408, 97.123)	.003
Control, mean (SD)	65.23 (12.18)	62.09 (10.18)	66.43 (9.14)	—^c^	—	—	—	—	—
*t* test (*df*)	−0.860 (69)	2.141 (69)	1.058 (69)	—	—	—	—	—	—
*P value*	.39	.036	.29	—	**—**	—	—	—	—
Cohen *d*	−0.20	0.51	0.25	—	—	—	—	—	—
Tenacity									
Intervention, mean (SD)	31.31 (5.80)	34.11 (7.43)	34.42 (5.89)	4.305 (1.419, 97.920)	.03	0.173 (1, 69)	.68	7.757 (1.419, 97.920)	.003
Control, mean (SD)	33.31 (6.87)	31.26 (6.00)	33.66 (4.89)	—	—	—	—	—	—
*t* test (*df*)	−1.332 (69)	1.778 (69)	0.590 (69)	—	—	—	—	—	—
*P* value	.19	.08	.56	**—**	—	—	—	—	—
Cohen *d*	−0.31	0.42	0.14	—	—	—	—	—	—
Strength									
Intervention, mean (SD)	22.14 (3.12)	19.31 (3.78)	23.67 (3.63)	112.710 (1.638, 113.004)	<.001	0.538 (1, 69)	.47	2.070 (1.638, 113.004)	.14
Control, mean (SD)	22.26 (4.19)	18.11 (2.99)	23.06 (3.66)	—	—	—	—	—	—
*t* test (*df*)	−0.135 (69)	1.471 (69)	0.704 (69)	—	—	—	—	—	—
*P* value	.89	.15	.48	—	—	—	—	—	—
Cohen *d*	−0.03	0.35	0.17	—	—	—	—	—	—
Optimism									
Intervention, mean (SD)	9.58 (2.03)	10.47 (1.72)	10.67 (1.43)	4.570 (1.626, 112.228	.02	2.466 (1, 6)	.12	5.366 (1.626, 112.228)	.01
Control, mean (SD)	9.66 (2.39)	9.43 (2.10)	9.71 (1.90)	—	—	—	—	—	—
*t* test (*df*)	−0.140 (69)	2.294 (69)	2.386 (69)	—	—	—	—	—	—
*P* value	.89	.03	.02	**—**	—	—	—	—	—
Cohen *d*	−0.04	0.54	0.57	—	—	—	—	—	—

a CD-RISC: Connor-Davidson Resilience Scale.

b Cohen *d* was calculated for between-group comparisons at each time point using the pooled SD. Positive values indicate higher scores in the intervention group, whereas negative values indicate higher scores in the control group.

c Not applicable.

**Table 3.** Comparison of HADS^a^ scores between the two groups (mean and SD)^b^.

Items and groups	T0	T1	T2	Statistics					
				Time effect		Group effect		Group×time	
				*F* test (*df*)	*P* value	*F* test (*df*)	*P* value	*F* test (*df*)	*P* value
HADS									
Intervention group, mean (SD)	14.44 (2.16)	11.78 (1.48)	9.69 (1.51)	263.588 (1.613, 111.281)	<.001	42.059 (1, 69)	<.001	68.906 (1.613, 111.281)	<.001
Control group, mean (SD)	14.57 (1.60)	15.86 (1.54)	12.03 (1.42)	—^c^	—	—		—	—
*t* test (*df*)	−0.283 (64.462)	−11.411 (69)	−6.700 (69)	—	—	—		—	—
*P* value	.78	<.001	<.001	—	—	—		—	—
Cohen *d*	−0.07	−2.70	−1.60	—	—	—		—	—
Anxiety									
Intervention group, mean (SD)	7.75 (1.71)	5.97 (0.74)	4.83 (1.06)	193.779 (1.689, 116.522)	<.001	4.096 (1, 69)	.047	35.174 (1.689, 116.522)	<.001
Control group, mean (SD)	7.37 (1.37)	7.86 (1.33)	4.89 (1.30)	—	—	—	—	—	—
*t* test (*df*)	1.025 (69)	−7.353 (52.693)	−0.187 (69)	—	—	—	—	—	—
*P* value	.31	<.001	.85	—	—	—	—	—	—
Cohen *d*	0.24	−1.76	−0.05	—	—	—	—	—	—
Depression									
Intervention group, mean (SD)	6.69 (1.86)	5.81 (1.28)	4.86 (1.18)	44.896 (1.755, 121.122)	<.001	29.499 (1, 69)	<.001	39.552 (1.755, 121.122)	<.001
Control group, mean (SD)	7.20 (1.41)	8.00 (1.39)	7.14 (1.14)	—	—	—	—	—	—
*t* test (*df*)	−1.286 (69)	−6.906 (69)	−8.298 (69)	—	—	—	—	—	—
*P* value	.20	<.001	<.001	—	—	—	—	—	—
Cohen *d*	−0.31	−1.64	−1.96	—	—	—	—	—	—

a HADS: Hospital Anxiety and Depression Scale.

b Cohen *d* was calculated for between-group comparisons at each time point using the pooled SD. Negative values indicate lower scores in the intervention group. For HADS scores, lower values indicate lower levels of anxiety and depress.

c Not applicable.

**Table 4.** Comparison of SSRS^a^ scores between the two groups (mean and SD)^b^.

Items and groups	T0	T1	T2	F-value (*P* value)					
				Time effect		Group effect		Group×time	
				*F* test (*df*)	*P* value	*F* test (*df*)	*P* value	*F* test (*df*)	*P* value
SSRS score									
Intervention, mean (SD)	45.25 (5.63)	46.86 (4.34)	46.97 (4.16)	21.091 (1.094, 75.500)	<.001	2.981 (1, 69)	.09	0.890 (1.094, 75.500)	.36
Control, mean (SD)	47.74 (6.43)	48.89 (5.76)	48.83 (5.29)	—^c^	—	—	—	—	—
*t* test (*df*)	−1.739 (69)	−1.676 (69)	−1.646 (69)	—	—	—	—	—	—
*P* value	.09	.098	.10	—	—	—	—	—	—
Cohen *d*	−0.41	−0.40	−0.39	—	—	—	—	—	—
Subjective support									
Intervention, mean (SD)	27.36 (3.57)	28.14 (3.07)	28.22 (2.90)	7.483 (1.072, 73.953)	.007	0.461 (1, 69)	.50	3.366 (1.072, 73.953)	.07
Control, mean (SD)	28.34 (3.80)	28.46 (3.69)	28.54 (3.45)	—	—	—	—	—	—
*t* test (*df*)	−1.122 (69)	−0.395 (69)	−0.424 (69)	—	—	—	—	—	—
*P* value	.27	.69	.67	—	—	—	—	—	—
Cohen *d*	−0.27	−0.09	−0.10	—	—	—	—	—	—
Objective support									
Intervention, mean (SD)	11.56 (2.36)	11.61 (1.83)	11.64 (1.84)	4.10 (1.227, 84.666)	.04	3.709 (1, 69	.06	2.776 (1.227, 84.666)=	.09
Control, mean (SD)	12.11 (2.17)	12.80 (2.27)	12.60 (1.97)	—	—	—	—	—	—
*t* test (*df*)	−1.039 (69)	−2.434 (69)	−2.124 (69)	—	—	—	—	—	—
*P* value	.30	.02	.04	—	—	—	—	—	—
Cohen *d*	−0.24	−0.58	−0.50	—	—	—	—	—	—
Support utilization									
Intervention, mean (SD)	6.92 (1.71)	7.11 (1.60)	7.11 (1.60)	0.764 (1.044, 72.062	.39	2.951 (1, 69)	.09	0.764 (1.044, 72.062)	.39
Control, mean (SD)	7.66 (1.59)	7.63 (1.46)	7.69 (1.41)	—	—	—	—	—	—
*t* test (*df*)	−1.887 (69)	−1.424 (69)	−1.604 (69)	—	—	—	—	—	—
*P* value	.03	.16	.11	—	—	—	—	—	—
Cohen *d*	−0.45	−0.34	−0.38	—	—	—	—	—	—

a SSRS: Social Support Rating Scale.

b Cohen d was calculated for between-group comparisons at each time point using the pooled SD. Positive values indicate higher scores in the intervention group, whereas negative values indicate higher scores in the control group.

c Not applicable.

**Table 5.** Comparison of BCSSS^a^ scores between the two groups (mean and SD)^b^.

Groups	T0	T1	T2	Statistics					
				Time effect		Group effect		Group×time	
				*F* test (*df*)	*P* value	*F* test (*df*)	*P* value	*F* test (*df*)	*P* value
Intervention, mean (SD)	39.25 (4.29)	42.36 (3.59)	43.22 (3.17)	40.838 (1.782, 122.981)	<.001	1.244 (1, 69)	.27	20.964 (1.782, 122.981)	<.001
Control, mean (SD)	40.29 (5.77)	39.23 (4.90)	42.06 (4.22)	—^c^	—	—	—	—	—
*t* test (*df*)	−0.860 (69)	3.083 (69)	1.317 (69)	—	—	—	—	—	—
*P* value	0.393	0.003	0.192	—	—	—	—	—	—
Cohen *d*	−0.20	0.73	0.31	—	—	—	—	—	—

a BCSSS: Breast Cancer Survivor Self-Efficacy Scale.

b Cohen *d* was calculated for between-group comparisons at each time point using the pooled SD. Positive values indicate higher scores in the intervention group, whereas negative values indicate higher scores in the control group.

c Not applicable.

**Table 6.** Comparison of FACT-B^a^ scores between the two groups (mean and SD)^b^.

Items and groups	T0	T1	T2	Statistics					
				Time effect		Group effect		Group×time	
				*F* test (*df*)	*P* value	*F* test (*df*)	*P* value	*F* test (*df*)	*P* value
FACT-B scores									
Intervention, mean (SD)	84.17 (13.96)	92.25 (12.55)	94.25 (11.03)	17.737 (1.023, 70.579	<.001	0.311 (1, 69)	.58	0.874 (1.023, 70.579)	.36
Control, mean (SD)	84.77 (19.89)	90.09 (12.11)	91.11 (11.41)	—^c^	—	—	—	—	—
*t* test (*df*)	−0.149 (69)	0.739 (69)	1.178 (69)	—	—	—	—	—	—
*P* value	.88	.46	.24	—	—	—	—	—	—
Cohen *d*	−0.04	0.18	0.28	—	—	—	—	—	—
Physical status									
Intervention, mean (SD)	17.06 (4.76)	18.61 (3.33)	19.19 (2.45)	10.459 (1.090, 75.234	.001	3.885 (1, 69	.05	0.036 (1.090, 75.234	.87
Control, mean (SD)	15.74 (5.76)	17.06 (3.07)	17.71 (2.35)	—	—	—	—	—	—
*t* test (*df*)	1.048 (69)	2.043 (69)	2.600 (69)	—	—	—	—	—	—
*P* value	.30	.045	.01	**—**	—	—	—	—	—
Cohen *d*	0.25	0.48	0.62	—	—	—	—	—	—
Social status									
Intervention, mean (SD)	17.94 (3.85)	19.81 (2.65)	19.89 (2.61)	11.370 (1.005, 69.350	.001	1.907 (1, 69)	.17	0.035(1.005, 69.350	.85
Control, mean (SD)	19.09 (4.62)	20.80 (4.20)	20.80 (4.20)	—	—	—	—	—	—
*t* test (*df*)	−1.129 (66.171)	−1.190 (57.097)	−1.095 (56.518)	—	—	—	—	—	—
*P* value	.26	.24	.28	—	—	—	—	—	—
Cohen *d*	−0.27	−0.28	−0.26	—	—	—	—	—	—
Emotional status									
Intervention, mean (SD)	14.83 (4.29)	15.89 (3.73)	16.06 (3.50)	9.041 (1.023, 70.588)	.003	0.746 (1, 69)	.39	0.216 (1.023, 70.588	.65
Control, mean (SD)	15.26 (5.10)	16.74 (3.18)	16.89 (3.03)	—	—	—	—	—	—
*t* test (*df*)	−0.380 (69)	−1.037 (69)	−1.068 (69)	—	—	—	—	—	—
*P* value	.71	.30	.29	—	—	—	—	—	—
Cohen *d*	−0.09	−0.24	−0.25	—	—	—	—	—	—
Functional status									
Intervention, mean (SD)	12.36 (4.82)	14.61 (4.75)	15.03 (4.32)	12.288 (1.028, 70.963)	.001	1.217 (1, 69)	.27	1.273 (1.028, 70.963)	.26
Control, mean (SD)	12.23 (4.29)	13.43 (3.15)	13.57 (2.94)	—	—	—	—	—	—
*t* test (*df*)	0.122 (69)	1.239 (60.923)	1.664 (61.887)	—	—	—	—	—	—
*P* value	.90	.22	.10	—	—	—	—	—	—
Cohen *d*	0.03	0.29	0.39	—	—	—	—	—	—
Additional concerns									
Intervention, mean (SD)	21.97 (4.12)	23.33 (3.28)	24.08 (2.91)	2.686 (1.062, 73.303)	.10	1.341 (1, 69)	.25	5.219 (1.062, 73.303)	.02
Control, mean (SD)	22.46 (4.76)	22.06 (3.76)	22.14 (3.81)	—	—	—	—	—	—
*t* test (*df*)	−0.460 (69)	1.526 (69)	=2.408 (63.682)	—	—	—	—	—	—
*P* value	.65	.13	.02	—	—	—	—	—	—
Cohen *d*	−0.11	0.36	0.57	—	—	—	—	—	—

a FACT-B: Functional Assessment of Cancer Therapy-Breast.

b Cohen *d* was calculated for between-group comparisons at each time point using the pooled SD. Positive values indicate higher scores in the intervention group, whereas negative values indicate higher scores in the control group.

c Not applicable.

### Psychological Resilience

**Figure 3.**
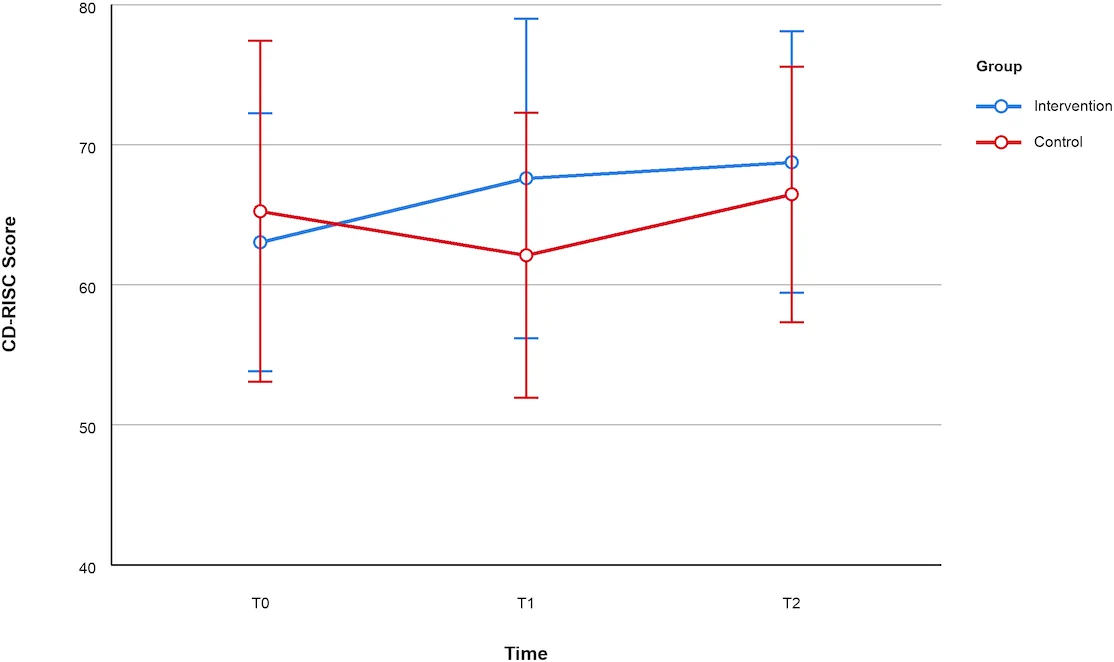
Connor-Davidson Resilience Scale (CD-RISC) scores over time by group. Error bars indicate SDs.

No statistically significant differences were observed in CD-RISC total or subscale scores between the two groups at baseline (T0). The time effect was statistically significant for the total resilience score and all subscale scores in both groups (*P*<.05). The between-group effect was not statistically significant for the total resilience score or any subscale scores (*P*>.05). A statistically significant time×group interaction effect was found for the total resilience score as well as the tenacity and optimism subscales (*P*<.05; Table 2). As shown in Figure 3, the total resilience score of the intervention group exhibited an upward trend over time, whereas the control group showed an initial decline followed by an increase. For the CD-RISC total score, the between-group effect size was moderate at T1 (Cohen *d*=0.51) and small at T2 (Cohen *d*=0.25).

### Anxiety and Depression

**Figure 4.**
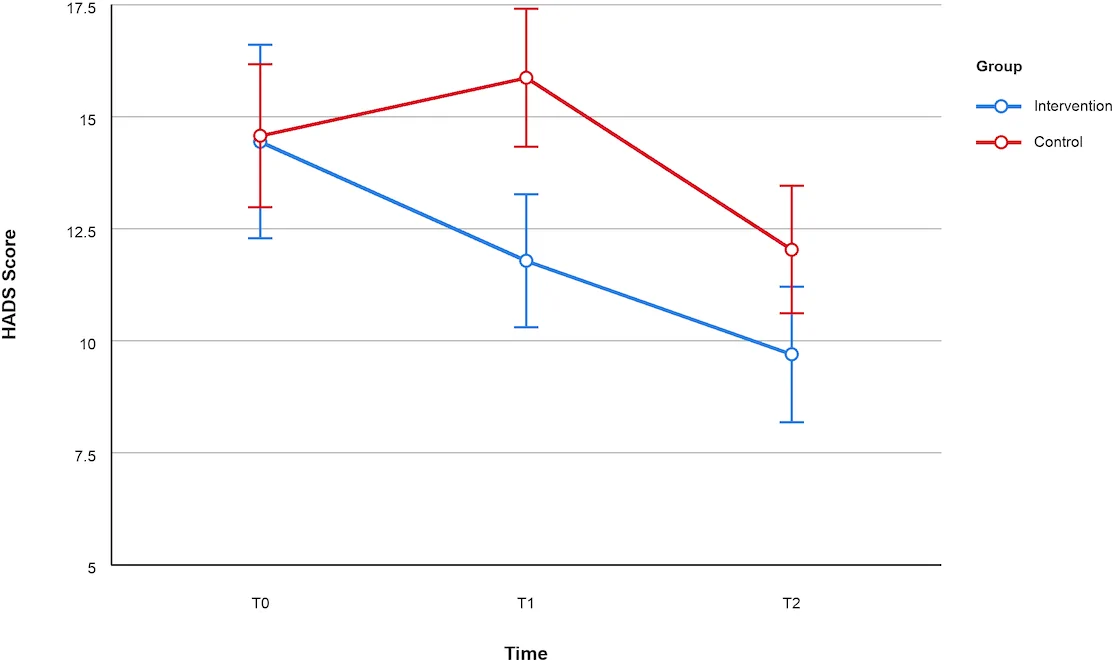
Hospital Anxiety and Depression Scale (HADS) scores over time by group. Error bars indicate SDs.

The results showed no statistical differences in the total HADS scores or the individual anxiety and depression subscale scores at T0 between the two groups. The time effect, group effect, and group×time interaction effect for both the total HADS scores and the individual anxiety and depression subscale scores were all statistically significant (*P*<.05). Details are presented in Table 3. As shown in Figure 4, the intervention group exhibited a declining trend in total HADS scores over time, whereas the control group showed an initial increase followed by a subsequent decrease. For the total HADS score, the between-group effect sizes were large at both T1 (Cohen *d*=−2.70) and T2 (Cohen *d*=−1.60), indicating lower overall anxiety and depression scores in the intervention group.

### Social Support

**Figure 5.**
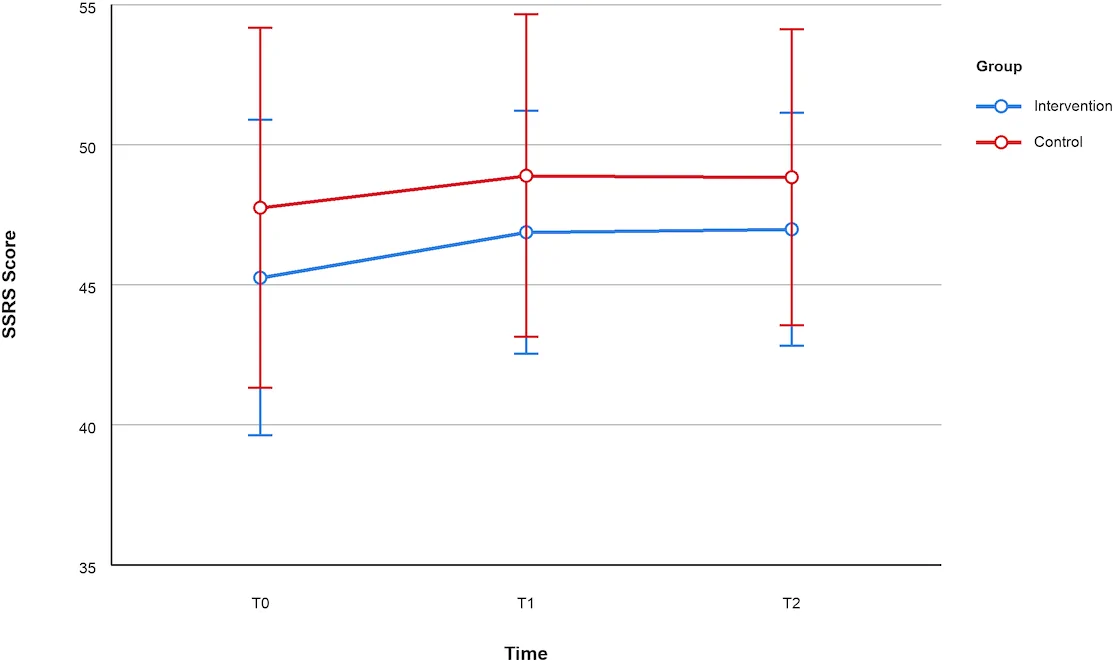
Social Support Rating Scale (SSRS) scores over time by group. Error bars indicate SDs.

The results showed no statistically significant differences in SSRS and subscale scores between the two groups at T0. The time effect was statistically significant for the total SSRS score, as well as for subjective and objective support subscale scores (*P<*.05). However, neither the group effect nor the group×time interaction effect reached statistical significance for the total SSRS score or any subscale scores (*P*>.05; Table 4). As shown in Figure 5, the intervention group exhibited a progressive increase in social support level over time, whereas the control group showed an initial rise followed by a decline. For the total SSRS score, the between-group effect sizes remained small and relatively stable at T0 (Cohen *d*=−0.41), T1 (Cohen *d*=−0.40), and T2 (Cohen *d*=−0.39).

### Self-Efficacy

**Figure 6.**
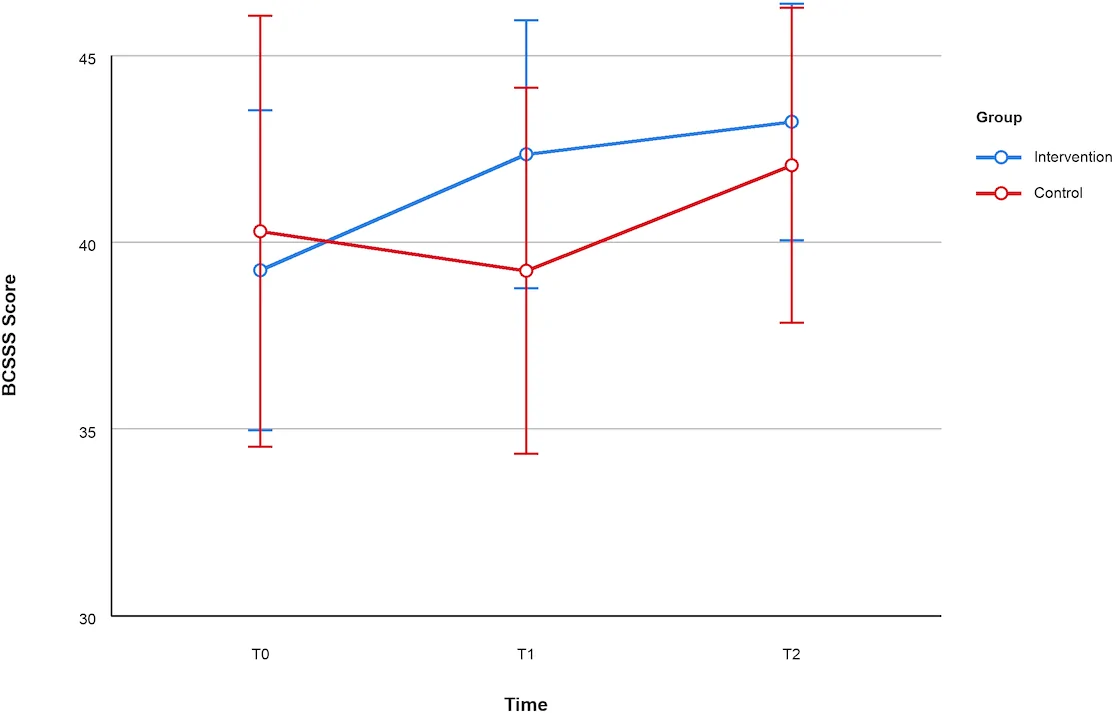
Breast Cancer Survivor Self-Efficacy Scale (BCSSS) scores over time by group. Error bars indicate SDs.

As shown in Table 5, the analysis revealed no statistically significant between-group difference in BCSSS scores at baseline (T0). The time effect and group×time interaction effect for the BCSSS scores in both groups were statistically significant (*P*<.05). In contrast, the group effect for BCSSS scores was not statistically significant (*P*>.05). As shown in Figure 6, the intervention group exhibited a progressive increase in BCSSS scores over time, whereas the control group showed an initial decline followed by subsequent improvement. For the BCSSS score, the between-group effect size was 0.73 at T1 and decreased to 0.31 at T2.

### Quality of Life

**Figure 7.**
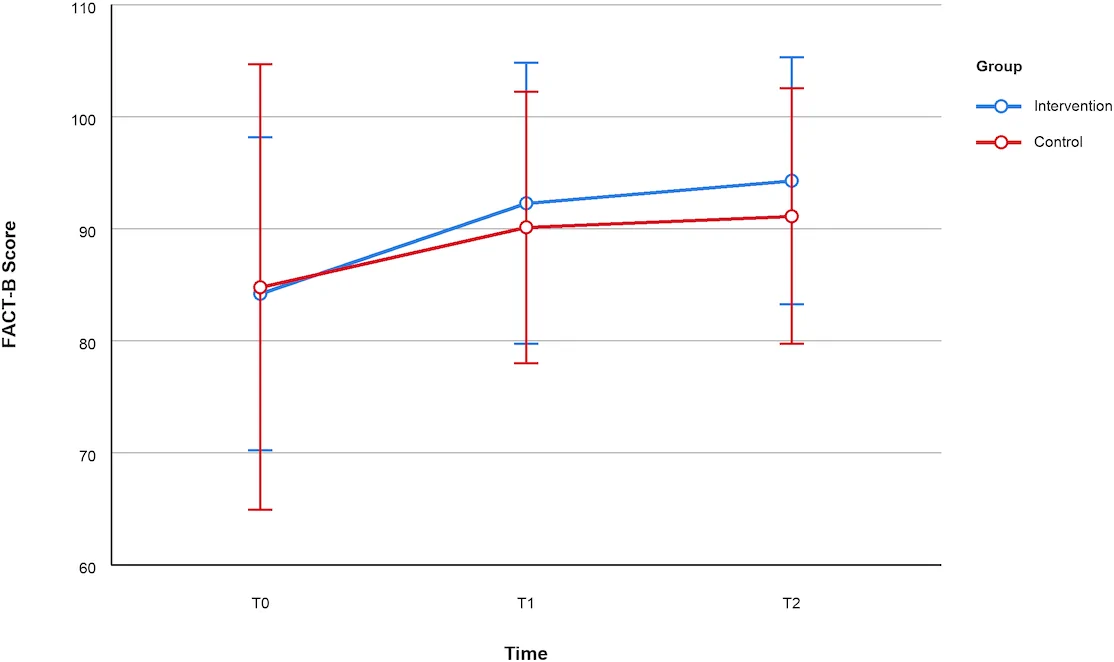
FACT-B scores over time by group. Error bars indicate SDs.

The results showed that there were no statistically significant differences in FACT-B scores or dimension scores between the two groups at T0. The time effect was statistically significant for the FACT-B scores and dimension scores (except for the additional concerns dimension) in both groups (*P*<.05). However, there were no statistically significant differences in the group effect for the FACT-B scores and dimension scores between the two groups (*P*>.05). A significant group×time interaction effect was observed only in the additional concerns dimension (*P*<.05). Details are presented in Table 6. As shown in Figure 7, the total FACT-B scores of both groups exhibited an upward trend over time. For the total FACT-B score, the between-group effect sizes remained small at T0 (Cohen *d*=−0.04), T1 (Cohen *d*=0.18), and T2 (Cohen *d*=0.28).

## Discussion

### Overview

This study evaluated a digital mindfulness intervention developed based on the RM-BC model, which was designed for home-based recovery of postoperative patients with breast cancer during chemotherapy intervals. The findings showed distinct patterns across the measured outcomes. The intervention group demonstrated a more favorable recovery trajectory in psychological resilience and self-efficacy, with significant between-group differences observed at the early follow-up but not maintained at the 6-month follow-up. Anxiety and depression showed more sustained improvement, with lower HADS scores in the intervention group at both follow-up points. In contrast, no clear statistically significant between-group differences were observed in social support or quality of life, although the direction of change was generally favorable. These findings suggest that the intervention may be particularly useful in buffering early psychological stress and supporting emotional adjustment during the vulnerable period of home recovery between chemotherapy cycles.

### Effects on Psychological Resilience

The repeated-measures ANOVA for CD-RISC total scores revealed a nonsignificant effect of group (*F_1,69_*=0.751, *P*=.389), indicating that the overall mean level of psychological resilience across the three time points did not differ significantly between the intervention and control groups. However, a statistically significant group×time interaction effect was observed (*F_1.408,97.123_*=7.595, *P*=.003), demonstrating that the trajectories of psychological resilience differed significantly between the two groups. As shown in Figure 3, the control group exhibited an initial decline in resilience at T1 followed by a rebound at T2. This pattern may reflect the natural restoration process of psychological resilience after the trauma of disease and treatment. In marked contrast, the intervention group did not exhibit such an initial decline. Instead, its resilience scores showed continuous improvement from baseline onward. Moreover, compared with the control group, the intervention group demonstrated a significantly faster rate of recovery in psychological resilience after the intervention (Figure 3). Specifically, at T1, the CD-RISC total score in the intervention group (mean 67.58, SD 11.41) was already higher than that in the control group (mean 62.09, SD 10.18); at T2, the intervention group (mean 68.75, SD 9.34) continued to show higher scores than the control group (mean 66.43, SD 9.14). These differences suggest that while the RM-BC model-based digital intervention did not yield a sustained absolute advantage in overall mean scores, it played a crucial protective role in preventing the commonly observed decline in psychological resilience during the early transitional phase of postoperative chemotherapy and in accelerating the trajectory of resilience recovery. At T1, the between-group difference in CD-RISC total scores corresponded to a moderate effect size (Cohen *d*=0.51). At T2, the effect size decreased to a small magnitude (Cohen *d*=0.25), which was consistent with the reduced between-group difference observed at the 6-month follow-up. However, we did not identify a validated minimal clinically important difference for the CD-RISC in postoperative patients with breast cancer during chemotherapy intervals. Therefore, although the short-term difference may be of potential clinical relevance, its clinical meaningfulness should be interpreted cautiously.

The mechanisms through which the digital intervention promoted recovery in psychological resilience may mainly involve two interrelated pathways. First, the intervention guided and encouraged patients to engage in regular mindfulness practice at home, including yoga, meditation, and breathing exercises. Sustained mindfulness practice may help patients internalize mindfulness as a personal coping strategy, thereby improving emotion regulation, alleviating physical discomfort, and managing intrusive thoughts [32,33]. These effects may, in turn, reduce the “risk factors” described in the RM-BC model [19]. Second, patients shared their experiences of mindfulness practice, symptom management, and rehabilitation through the interactive module of the digital platform. This process not only provided emotional validation and social support for participants who shared their stories but also allowed others to derive hope and motivation from their peers’ positive experiences. Such online interaction may have strengthened the “protective factors” in the RM-BC model, including social support, self-efficacy, and hope. The combined effect of reducing risk factors and enhancing protective factors may have helped offset the decline in psychological resilience that could otherwise be triggered by early stressors during the postoperative chemotherapy interval, while also supporting a steady improvement in resilience among patients in the intervention group.

In summary, the resilience trajectory observed in the control group appears to reflect the natural pattern of psychological recovery after trauma, whereas the sustained linear improvement observed in the intervention group highlights the potential value of the digital mindfulness intervention during the early recovery phase of the postoperative chemotherapy interval. Its benefit may not lie merely in raising the upper limit of resilience, but rather in buffering the impact of early stressors through timely intervention. In this way, the intervention may help preserve the continuity and stability of psychological recovery and protect patients from stress-related disruptions during this vulnerable period [34].

### Effects on Anxiety and Depression

This study found that HADS scores in the intervention group were lower than those in the control group at both T1 (mean 11.78, SD 1.48 vs mean 15.86, SD 1.54) and T2 (mean 9.69, SD 1.51 vs mean 12.03, SD 1.42). This suggests that the digital intervention based on the RM-BC model may help alleviate anxiety and depression among patients with breast cancer during the postoperative chemotherapy interval. This finding is generally consistent with the results reported by Salman et al [35]. Figure 4 further shows that the longitudinal trends in HADS scores differed between the two groups. In the control group, anxiety/depression scores increased at T1 and then partially decreased at T2, although they remained higher than those in the intervention group. In contrast, HADS scores in the intervention group showed a continuous downward trend throughout the follow-up period. Notably, the between-group difference in the anxiety subscale score at T2 did not reach statistical significance. This may be related to the gradual psychological adaptation of patients in the control group over time and the partial relief of treatment-related stress, suggesting that the between-group advantage of the digital intervention in reducing anxiety symptoms may have diminished during the later follow-up period.

Overall, the decreasing trend in HADS scores was opposite to the increasing trend in CD-RISC scores, suggesting a possible association between the alleviation of anxiety and depression and the improvement of psychological resilience. In other words, the digital intervention may have promoted resilience recovery not only by enhancing patients’ positive psychological resources but also by reducing negative emotions such as anxiety and depression, thereby mitigating the interference of early stressors with the psychological recovery process. This finding further supports the interpretation of resilience recovery trajectories from the perspective of negative emotional outcomes.

Mindfulness-based interventions emphasize purposeful and nonjudgmental awareness of present-moment experiences, thereby cultivating attentional regulation and emotional adjustment. In this study, with the guidance of meditation audio materials provided through the mini-program, patients learned to anchor their attention to breathing during seated meditation, breathing exercises, and yoga practice. They were encouraged to observe changes in breathing rhythm, muscle tension, and bodily sensations, while noticing chemotherapy-related emotional fluctuations with a less reactive attitude. Meanwhile, mindfulness body scanning helped patients extend this nonjudgmental awareness to whole-body experiences, enabling them to consciously identify, accept, and adaptively respond to areas of tension and discomfort during yoga postures coordinated with breathing and movement [36]. Such mind-body integrative practice may help patients better cope with physical discomfort and psychological distress during the postoperative chemotherapy interval and approach treatment-related challenges in a calmer and more proactive manner.

In addition, regular practice of the mindfulness course may not only cultivate present-moment awareness, acceptance, and emotional regulation, but also provide a structured, low-intensity, home-based form of physical activity through mindful yoga [37]. Therefore, the intervention may have exerted its effects through dual pathways involving psychological regulation and physical activity. On the one hand, mindfulness practice may help reduce rumination, emotional reactivity, and threat perception; on the other hand, yoga and breathing exercises may promote physical relaxation, relieve muscle tension, and improve patients’ tolerance of bodily discomfort. These synergistic physiological and psychological effects may be an important reason for the sustained improvement in anxiety and depression observed in the intervention group.

### Effects on Social Support

The results showed that there were no statistically significant differences in SSRS scores between the two groups at any time point, suggesting that the digital intervention had a relatively limited effect on improving social support among patients with breast cancer during the postoperative chemotherapy interval. Compared with individual-level psychological variables such as anxiety, depression, and self-efficacy, the formation and enhancement of social support may depend more heavily on long-term, stable, and high-quality interpersonal networks. Although short-term digital interaction can provide some informational support, experience sharing, and emotional companionship, it may be insufficient to significantly change patients’ overall social support status within a relatively short follow-up period.

This finding may also be related to the inherent limitations of online interaction. Social support includes not only informational and experiential support but also sustained emotional responsiveness, relational trust, and real-life companionship. For patients with breast cancer during the postoperative chemotherapy interval, social support may come not only from peer communication but also from family caregiving, communication with healthcare professionals, and continuous companionship in daily life. Therefore, relying solely on an online interactive module may be insufficient to substantially change the overall level of social support reflected by SSRS scores within a short period.

Nevertheless, social support remains an important protective factor in the RM-BC model and an important foundation for improving psychological resilience, self-efficacy, and quality of life. In this study, peer experience sharing and emotional support provided through the interactive module may have helped alleviate patients’ disease-related isolation to some extent and provided them with rehabilitation references and psychological encouragement. However, such support may have been more situational and supplementary in nature, and may not have been sufficient to translate into significant improvements in social support scale scores.

Future studies could further optimize the interactive design of digital platforms by incorporating nurse-led thematic discussions, peer group communication, regular online group activities, or a combination of online and face-to-face support. These strategies may enhance the continuity, emotional depth, and intensity of interaction. By improving the quality and continuity of online interaction, digital interventions may more effectively promote patients’ social support.

### Effects on Self-Efficacy

Repeated-measures ANOVA of BCSSS scores showed a significant time effect (*F_1.782,122.981_*=40.84, *P*<.001), but the main effect of group was not statistically significant (*F_1,69_*=1.24, *P*=.27). This indicates that self-efficacy changed over time in both groups, but the overall mean self-efficacy scores across the three time points did not differ significantly between the intervention and control groups. However, the single-time-point between-group comparison at 3 months post intervention (T1) showed that the self-efficacy score in the intervention group was significantly higher than that in the control group (mean 42.36, SD 3.59 vs mean 39.23, SD 4.90; *P*=.003). This finding suggests that the effect of the digital intervention on self-efficacy may have been most evident in the early postintervention period. By the 6-month follow-up, as patients in the control group gradually adapted to the treatment and recovery process, the between-group difference may have weakened, resulting in a nonsignificant overall group effect.

In terms of longitudinal trends, Figure 6 shows that self-efficacy scores in the intervention group continued to increase after receiving the digital intervention, whereas the control group showed a pattern of initial decline followed by recovery. This trajectory was broadly consistent with the pattern of psychological resilience observed in this study, suggesting that the digital intervention may have provided support during the early postoperative chemotherapy interval and helped patients build confidence in disease management, symptom coping, and rehabilitation more rapidly. In other words, the value of the intervention may not necessarily lie in producing a long-term and stable absolute between-group advantage, but rather in buffering the early decline in self-efficacy and facilitating the earlier restoration of active coping capacity.

According to Bandura’s self-efficacy theory, self-efficacy is shaped mainly by mastery experiences, vicarious experiences, verbal persuasion, and physiological and emotional states [38]. In this study, the mindfulness course delivered through the WeChat mini-program provided patients with continuous and practical self-management exercises. Through regular breathing exercises, meditation, and yoga practice, patients may have gradually accumulated mastery experiences, such as “I can actively regulate my own state” and “I can cope with treatment-related discomfort.” These sustained practice experiences may have strengthened patients’ confidence in their self-management abilities.

At the same time, peer sharing in the interactive module may have provided patients with vicarious experiences and emotional encouragement. By reading or participating in the sharing of rehabilitation experiences from peers in similar situations, patients could observe how others persisted in practice, managed symptoms, and gradually recovered, thereby enhancing their own confidence and expectations for recovery. In addition, the check-in reminders and practice records in the platform may have reinforced patients’ sense of participation and accomplishment, allowing them to visually track their continued practice. These factors may jointly explain why self-efficacy was significantly higher in the intervention group than in the control group at T1.

### Effects on Quality of Life

This study found that FACT-B scores did not differ significantly between the two groups at T0, T1, or T2, indicating that the digital intervention did not produce a clear between-group difference in quality of life. However, the direction of the mean scores changed over time. At baseline, the control group had slightly higher FACT-B scores than the intervention group, whereas at both postintervention follow-up points, T1 and T2, the intervention group had higher mean FACT-B scores than the control group. Because these differences did not reach statistical significance, this trend should be interpreted cautiously. Nevertheless, it may suggest that the digital intervention based on the RM-BC model has a potential positive effect on quality of life among patients with breast cancer during the postoperative chemotherapy interval.

Quality of life reflects patients’ comprehensive subjective evaluation of their physical, psychological, and social functioning [39]. For patients with breast cancer receiving chemotherapy, quality of life is influenced by both treatment-related factors and psychosocial factors, including anxiety, depression, psychological resilience, and social support [40,41]. Therefore, improvement in quality of life is unlikely to be achieved rapidly through a single intervention component alone; rather, it may require sustained and multidimensional psychological, behavioral, and social support.

Considering the overall pattern of outcomes in this study, the intervention group showed relatively favorable trends in psychological resilience, anxiety and depression, and self-efficacy, which may have provided a foundation for improved quality of life. First, at the psychological level, the continuous decrease in HADS scores in the intervention group suggests that anxiety and depression were alleviated. The reduction of negative emotions may improve patients’ subjective experience of the disease and treatment process, thereby potentially enhancing emotional functioning and overall quality of life. Second, in terms of positive psychological resources, improvements in psychological resilience and self-efficacy may have enhanced patients’ ability to cope with treatment stress, symptom fluctuations, and rehabilitation challenges.

In terms of intervention content, the WeChat mini-program provided support at both psychological and physical levels. Mindful breathing, meditation, and yoga practice may help relieve physical tension, improve fatigue, and enhance patients’ tolerance of bodily discomfort. At the same time, mindfulness practice may help patients regulate emotional responses and reduce excessive attention to symptoms and disease-related uncertainty. The interactive module provided a degree of social connection and rehabilitation reference through peer experience sharing and emotional support. Although SSRS scores did not show significant between-group differences in this study, online interaction may still have served as a supplementary source of support, helping patients obtain experiential references and psychological encouragement.

It should be noted that improvement in quality of life is usually a comprehensive and gradual process, and a longer period of sustained intervention may be required before significant changes can be detected in scale scores. In this study, FACT-B scores in the intervention group were higher than those in the control group at both T1 and T2, but the differences were not statistically significant. This suggests that the digital intervention may have potential benefits for quality of life, but its effect intensity, intervention duration, or sample size may still have been insufficient to generate clear between-group differences. Future studies may consider extending the intervention period, optimizing the quality of support provided through the interactive module, and further strengthening comprehensive intervention components targeting symptom management, social support, and self-efficacy, so as to more fully evaluate the long-term effects of digital interventions on quality of life.

### Study Limitations

This study has several limitations. First, this was a single-center quasi-experimental study. Participants were allocated at the ward level rather than through individual randomization. This approach may have reduced contamination among patients within the same ward. However, it could not fully eliminate selection bias, clustering effects, or differences in ward environments and nursing practices. Therefore, the strength of causal inference remains limited. The generalizability of the findings should also be interpreted with caution. Future studies should adopt a multicenter randomized controlled design. Statistical analyses should also account for potential clustering effects.

Second, the sample size was estimated based on psychological resilience as the primary outcome. However, the study may still have had limited power to detect small between-group differences in secondary outcomes, such as social support and quality of life. Therefore, nonsignificant findings with favorable trends should be interpreted cautiously. In addition, some intervention effects appeared to diminish at the 6-month follow-up. This suggests that the digital intervention may be more effective in buffering early stress during home recovery between postoperative chemotherapy cycles. However, the durability of these effects requires further investigation. Future studies could increase the sample size and extend the follow-up period. Booster sessions or sustained support modules may also be incorporated to maintain intervention effects.

Finally, the implementation process and underlying mechanisms of the intervention were not fully evaluated. The digital platform provided mindfulness practice, interactive sharing, check-in reminders, and practice records. However, this study did not examine patients’ actual usage frequency, practice duration, or interactive engagement. It also did not explore how these engagement indicators were associated with changes in outcomes. Therefore, the relative contribution of each intervention component remains unclear. In addition, the proposed mechanism of reducing “risk factors” and enhancing “protective factors” was mainly based on the RM-BC model and observed outcome trends. It was not directly tested through mediation or path analysis. Future research should incorporate objective backend usage data from the platform. This would help examine intervention adherence, dose–response relationships, and mechanisms of action.

### Implications for Practice

The findings of this study support the potential use of the RM-BC model–based digital mindfulness intervention in continuing care for patients with breast cancer. This intervention may provide psychological support during home recovery between postoperative chemotherapy cycles.

During follow-up, nurses can assess psychological resilience, anxiety and depression, and self-efficacy as part of routine care. These assessments may help identify patients who need additional psychological support. Based on patients’ emotional status, physical condition, and functional recovery, nurses can provide individualized guidance on mindfulness practice and self-management.

In clinical practice, digital platforms such as WeChat mini-programs can be used to deliver structured mindfulness courses. They can also remind patients to practice regularly and provide opportunities for peer experience sharing. Interactive modules may offer supplementary emotional support during home-based recovery.

Compared with traditional one-time health education, this approach can extend nursing support beyond the hospital. It may help patients receive continuous, accessible, and low-burden psychological support between chemotherapy cycles. Future practice could integrate digital mindfulness interventions with symptom management, rehabilitation exercise guidance, and routine follow-up care. This may help develop a more comprehensive support model for postoperative rehabilitation in patients with breast cancer.

### Conclusion

This study evaluated a digital mindfulness intervention based on the RM-BC model, which was specially designed for the home-based recovery of postoperative patients with breast cancer during chemotherapy intervals.

The findings suggest that this intervention can help alleviate anxiety and depression. It also supported the early recovery of psychological resilience and self-efficacy. However, no clear statistically significant advantages were observed in social support or quality of life. The direction of change in these outcomes was generally favorable, but these findings should be interpreted with caution.

Overall, digital mindfulness interventions can help reduce the time, location, and physical burden associated with traditional face-to-face interventions. They may also serve as a useful supplement to psychological support and continuing care for patients with breast cancer during home-based recovery. Future studies with more rigorous designs are needed to verify the long-term effects and underlying mechanisms of this intervention and to inform the development of a comprehensive support model for postoperative rehabilitation in patients with breast cancer.

## Abbreviations

BCSSS: Breast Cancer Survivor Self-Efficacy Scale
CD-RISC: Connor-Davidson Resilience Scale
FACT-B: Functional Assessment of Cancer Therapy-Breast
HADS: Hospital Anxiety and Depression Scale
MBCR: Mindfulness-Based Cancer Recovery
PICC: peripherally inserted central catheter
RM-BC: Resilience Model for Breast Cancer
SSRS: Social Support Rating Scale
